# Stakeholders’ participation in planning and priority setting in the context of a decentralised health care system: the case of prevention of mother to child transmission of HIV programme in Tanzania

**DOI:** 10.1186/1472-6963-13-273

**Published:** 2013-07-12

**Authors:** Elizabeth H Shayo, Leonard EG Mboera, Astrid Blystad

**Affiliations:** 1 Department of Global Public Health and Primary Care, University of Bergen, P. O. Box 7804, 5020 Bergen, Norway; 2 National Institute for Medical Research, Tanzania, P.O. Box 9653, Dar es Salaam, Tanzania

**Keywords:** Planning, Priority setting, PMTCT, Decentralisation, Donor influence, Tanzania

## Abstract

**Background:**

In Tanzania, decentralisation processes and reforms in the health sector aimed at improving planning and accountability in the sector. As a result, districts were given authority to undertake local planning and set priorities as well as allocate resources fairly to promote the health of a population with varied needs. Nevertheless, priority setting in the health care service has remained a challenge. The study assessed the priority setting processes in the planning of the prevention of mother to child transmission of HIV (PMTCT) programme at the district level in Tanzania.

**Methods:**

This qualitative study was conducted in Mbarali district, south-western Tanzania. The study applied in-depth interviews and focus group discussions in the data collection. Informants included members of the Council Health Management Team, regional PMTCT managers and health facility providers.

**Results:**

Two plans were reported where PMTCT activities could be accommodated; the Comprehensive Council Health Plan and the Regional PMTCT Plan that was donor funded. As donors had their own globally defined priorities, it proved difficult for district and regional managers to accommodate locally defined PMTCT priorities in these plans. As a result few of these were funded. Guidelines and main priority areas of the Ministry of Health and Social Welfare (MoHSW) also impacted on the ability of the districts and regions to act, undermining the effectiveness of the decentralisation policy in the health sector.

**Conclusion:**

The challenges in the priority setting processes revealed within the PMTCT initiative indicate substantial weaknesses in implementing the Tanzania decentralisation policy. There is an urgent need to revive the strategies and aims of the decentralisation policy at all levels of the health care system with a view to improving health service delivery.

## Background

The introduction of primary health care implied a focus on enhancing health service provision at the grassroots. In fact, the ‘health for all’ concept of the 1978 Alma-Ata declaration became a prime policy bearer for the movement of bringing health services closer to the communities [1]. A grassroots and participatory focus, rather than a top-down approach, has since then gained recognition as a fundamental principle in attempts to ensure stakeholders involvement and fair priority setting in health care. The focus on ensuring broader participation in health care decision-making processes gained renewed attention and became more manifest in the early 1990s when many countries started implementing reforms as a strategy geared towards improving the performance of their health systems [2,3].

One of the major components of the health sector reforms in Tanzania was decentralisation through devolution and integration of the district health care services [4]. This reform included devolving political, administrative, financial, and personnel control from the central to the lower levels. In Tanzania, sectoral reforms were implemented in three phases between 1999 and 2001 involving all sectors. Each phase comprised a third of all local authorities [5]. Within the district health care system, the Council Health Management Team (CHMT) was formed, and was later followed by the District Health Boards (DHBs). By devolving decision-making to local authorities, decentralisation aimed at improving planning and accountability and ensuring that priority setting and decision-making processes were located close to the beneficiaries in the health sector [6,7]. Under this structuring process, the Ministry of Health and Social Welfare (MoHSW) remained with the responsibility of overall policy making, long-term and macro-planning, as well as of overall monitoring of the health sector. The districts, on their part, were given the authority to undertake local planning, allocate resources and manage district health services. The districts were also given the authority to supervise, monitor and evaluate the district’s programmes and interventions. With decentralisation, each district was moreover scheduled to develop its own Comprehensive Council Health Plan (CCHP) using block grants from the central government and basket funding (pooled donor funds). In this regard the CCHP has to be developed within the overall frames as outlined in the Essential Health Package produced by the MoHSW [8]. The distribution of funds to the districts is dependent on the CCHP guidelines and is furthermore based on the criteria of population size and special needs as developed and categorised at the national level [9]. The main expected benefits of decentralisation were local participation in the planning of the services, increased responsiveness to locally-experienced problems, and ultimately increased efficiency and quality. A few studies have been carried out to assess the status and the functioning of the decentralisation reform in Tanzania. These studies reveal that decentralisation has generally facilitated the establishment of health bodies and committees from national to community levels, a structure through which community priorities are to be brought up and later forwarded to the higher authorities. Studies by Maluka and Mubyaz found that well-defined structures aimed at ensuring grassroots participation have been established [10,11]. Some studies have also demonstrated the success of such structures in terms of community participation. These studies have, for example, demonstrated that communities identified and articulated their own problems, and through the available administrative structures found resources for implementing what they perceived to be relevant projects. Concrete success stories in the health system have been noted in the immunisation, malaria control and maternal and child health service provision. Active community participation has been described to contribute to such success, a success enhanced by increased empowerment in the population [12-14]. Other studies have shown how Tanzanian districts are preparing their own plans and how the ‘basket funds’ and ‘block grants’ are remitted to the district level based on these plans [8]. Basket funding in Tanzania remitted to the districts has facilitated the renovation of health facilities, the purchase of essential equipment and drugs to supplement inadequate kits. It has also been noted to increase opportunities for in-service-training of the existing staff, and facilitated the improvement of utilities such as water and electricity supply at many health facilities [15]. However other studies have documented substantial challenges within the decentralised health system. Although in principle health committees existed from the grass root level, in practice, some of the committees were largely in active [11,16]. Moreover, the problem has been compounded by the failure to include effectively community priorities in the district health plan [10]. These latter findings are problematic as decentralisation was expected to bring about more public deliberation on major, unsolved public policy problems, with people engaging in debates on how to utilise limited resources fairly to promote the health of a population with substantial and varied needs [17,18]. Generally, previous studies in Tanzania have demonstrated that this has not been the case in actuality [16,19].

While only few studies have been carried out on priority setting and planning processes within general health care in Tanzania [16], we wished however to scrutinise a more narrow-based initiative with the aim of giving a close look at the concrete priority setting and decision-making processes in the country’s health sector in the face of ongoing decentralisation processes. To date little is known about the present experiences in the decentralised planning processes pertaining to the programmes such as the prevention of mother to child transmission of HIV (PMTCT) programme. The objective of the present study was to assess the processes of priority setting in the planning process through a scrutinisation of the PMTCT programme implemented at district level in Tanzania in a decentralised health care system. More concretely the study explored the extent to which the needs and challenges identified and brought forward by key stakeholders at the regional, district and health facility levels pertaining to the PMTCT programme were incorporated into the health plans.

### A brief note on the PMTCT programme

The PMTCT programme was introduced in Tanzania in 2000. By 2007 its coverage was 32% [20]. In December 2009 the coverage had reached 78% (i.e. 3,626 of the 4,647 health facilities in the country were providing PMTCT services) [21]. PMTCT service provision has, as such at a very rapid pace become available in the majority of the country’s health facilities that offer Reproductive and Child Health (RCH) services. Priorities relating to the programme are to be covered by both the Communicable Disease Control (the second priority area of the Essential Health Package) under which HIV/AIDS-related activities fall, and under the RCH programme (the first priority area of the Essential Health Package) [8]. In 2007, the regional level was assigned under the PMTCT guidelines produced by the Ministry of Health with the responsibility of ensuring that the PMTCT services were included in the Comprehensive Council Health Plan produced at the district level [22]. The districts on their part remained with the tasks of implementing the PMTCT services by ensuring the availability of equipment, supplies and trained staff. The programme initially operated as a vertical programme, implying that funding was received directly from the donors by the implementers. However, with decentralisation all vertical programmes were to be subsumed within the larger health systems structure and were to operate horizontally. The ‘Prevention of Mother to Child Transmission of HIV’ (PMTCT) programme was in principle in 2011fully integrated into the reproductive and child health care services from national to health facility levels [23]. While PMTCT coverage has been a success story in general, it does not imply improved quality of care. The PMTCT programme has struggled with challenges of adherence and uptake, among others due to the demanding requirements of the changes of the guidelines [24-26] attributed by socio-cultural and economic in-appropriateness. To date little or nothing is known about the experiences with priority setting processes within the PMTCT programme being executed in the country.

## Methods

### Study setting

The study was conducted in 2011 in Mbarali district which is located in south-western Tanzania. Mbarali was chosen because it was the location of the study setting for the EU funded umbrella project for the present study, the Response for Accountable Priority Setting for Trust in Health System (REACT) [27]. The present sub-study, therefore, was loosely linked to the umbrella project. HIV was one of the major health domains studied under the auspices of REACT. The project was located in Mbeya partly due to the relatively high HIV prevalence in the region: 9% as compared to the national estimates of 5.1% in the country as a whole [28]. According to the 2002 National Population Census, Mbarali district had a population of 234,101 (114,738 male and 119,363 female) with an estimated annual growth rate of 3%. The main economic activities of the district are rice-farming and livestock-keeping. The district is served by both public and private health facilities. It has two hospitals, one health centre, and 43 dispensaries. The coverage of the health facilities providing PMTCT services in Mbarali at the time of the present study was 86% (32/37 health facilities). Since much have been documented on HIVAIDS in general, the PMTCT programme seemed to provide a particularly interesting case in a priority setting context due to the substantial focus that has been given to the programme during the last decade.

### Study design and recruitment of informants

The present study employed a qualitative study design using in-depth interviews (IDIs) and focus group discussions (FGDs) in the data collection. Purposive sampling was employed to recruit informants at the regional, district and health facility levels. All individuals being enrolled in the project had experience in either the management or the administrative aspects of the PMTCT programme. The main categories of informants included: 1) members of the regional team (three individuals: coordinators for HIV/AIDS, Reproductive and Child Health and for PMTCT; 2) members of the district management team (eight individuals including the District Medical Officer, District AIDS Coordinator, District Reproductive and Child Health Coordinator, District Nursing Officer, District pharmacist, District laboratory technologist, District home based care coordinator and the Tanzania Commission for AIDS focal person (TACAIDS); and 3) the PMTCT in-charges at the health facilities who were also managing the RCH services (10 individuals, five rural and five urban). The 22 participants in the two FGDs were recruited from two hospitals providing PMTCT services (one faith-based and one government hospital with 10 and 12 participants respectively) (Table 1). The FGDs were deemed appropriate because the hospital settings had many health workers performing PMTCT-related tasks both directly and indirectly (for example, the pharmacy, the laboratory, the maternity and reproductive child health clinic). The FGDs gave them an opportunity to participate in joint discussions to share their experiences of the implementation of the programme. The district HIV/AIDS focal person assisted in identifying the PMTCT in-charges from the selected health facilities while the District Medical Officer assisted in the identification of the informants at the regional and district levels. The selection criterion that the DMO used was ‘performing the PMTCT roles.’ Additional criteria factoring the rural–urban divide and the distance were applied to recruit PMTCT in-charges from different health facilities.

**Table 1:** Data collection and number of informants

**Data collection method**	**Number of IDIs/FGDs**	**Total**	**No. of informants**
In-depth interviews	Health facility PMTCT in-charges	10	**21**
	District managers	8	
	Regional managers	3	
	**Total**	**21**	
Focus Group discussions	Health facility workers involved in PMTCT	2	**22**
	**Total number of informants**		**43**

### Data collection

Individual in-depth interviews (IDIs) were carried out with the informants recruited at regional, district and health facility levels. The IDIs were supplemented by FGDs conducted with health care providers at the hospital level. All the informants had experience with the PMTCT services either as health workers or as administrators of the programme. The interviews and discussions were aimed at generating a broad-based understanding of the experiences with priority setting processes related to the PMTCT services. Since decentralisation is implemented at district level, an assessment of the manner in which local priorities were brought on board during the district planning process leading up to the CCHP was particularly focused. The district health plans are prepared annually and include all prioritised health-related activities of that respective year with financial allocations.

A research guide with similar themes was developed for each category of informants. Central questions in the guides included: *How are priorities related to PMTCT made at the district level? What criteria or procedures guide the priority setting processes? In what part of the budget are PMTCT activities accommodated? Have you ever been asked to provide inputs or submit your priority areas regarding PMTCT to the CHMT? What are the sources of funds for the planned PMTCT activities? What are the potential challenges you encounter in setting priorities related to the PMTCT?* The first author of this paper collected the data assisted by a competent social scientist. The last author provided guidance during the data collection process. The questions were addressed in an open manner, and the informants were allowed to speak at length without being subjected to interruptions. The interviews allowed for further probing to gain more information and insights on emerging issues relevant to the problem. All IDIs and FGDs were recorded with a digital recorder after obtaining consent from the informants. Three interviews were not voice-recorded due to technical problems. These interviews were recorded through rapid note taking. The FGDs had a mix of female and male participants as the discussion of the study topic was not perceived to be particularly sensitive to the gender composition of the groups. In fact, the informants did not find the topic particularly challenging to talk about; they seemed engaged in the topic and were highly articulate during both the interviews and the discussions. All interviews were carried out in Swahili, the national language and lingua franca for East and Central Africa.

### Data analysis

All the interviews and FGDs were transcribed verbatim by a competent transcriber. Preliminary analysis started right in the field. A more detailed analysis was carried out after the completion of the data collection period. Conventional content analysis [29] was employed in the analysis of the material. In our case the analysis process the following steps: the first author read all the transcripts and listened to the audios carefully to confirm the correctness of the transcriptions. This helped to achieve immersion and obtain a sense of the whole in the material [29]. The last author also reviewed a large number of the transcripts, and the content of the interviews was discussed in some detail between the two authors to ensure that the subsequent data analysis was in line with the research objective. The second stage entailed reading the transcripts slowly, highlighting the parts of the text pertaining to particular aspects of the present study. Relevant ‘codes’ briefly summing up the content, were created throughout the entire data set, implying the manual insertion of the ‘codes’ in the margins of the transcript pages. All the codes were sorted, listed and clustered into larger categories / themes addressing the key research questions as contained in the data collection guides as well as new themes emerging from the interviews. Quotes were employed that reflected the informants own words in a narrative report. Matrices were created by cutting and pasting related statements or dialogues into categories, making it possible to track patterns as well as nuances and ambiguities within the material. During the final phase, a systematic comparison of the content generated from both the IDIs and FGDs was carried out as well as between different levels of the informants. We believe such comparison of the data strengthened the reliability of the study findings and its trustworthiness, thus making the knowledge generated transferable to other similar settings. The study undertaking adhered to the guidelines on qualitative research as required by the *BMC Health Services Research Journal*.

### Ethical considerations

The study obtained ethical approval from the Medical Research Coordinating Committee of the National Institute for Medical Research of Tanzania (NIMR/HQ/R.8a/Vol. IX/1094). Permission to conduct the study was also obtained from regional and the district authorities. Also permission to publish the data was sought from the Director General of the National Institute for Medical Research, Tanzania. Before the interviews were initiated, oral informed consent was received from informants at all levels after the objectives of the study had been well explained to them. Privacy and confidentiality were strictly emphasized and maintained throughout the study. Informants were also assured of their right to withdraw from the discussion at any time they would wish.

## Results

### Experiences with planning and priority setting in PMTCT programme-related activities

The informants reported two plans in which locally- prioritised PMTCT activities could be accommodated: the Comprehensive Council Health Plan (CCHP) and the Regional Health Plan. Whereas the council health management team was responsible for preparation of the CCHP, the regional health management team was responsible to prepare the regional health plan. Within the decentralised model, the regional secretariats were tasked with reviewing the District Council Comprehensive Health Plans (CCHP) before they were further reviewed and approved by the District Full Council. The Regional Medical Officer, on his/her part was responsible for the approval of the regional plan before it was submitted to the funding agents who are mainly external partners. The prime planning and priority setting processes related to the PMTCT programme were, according to the regional and district informants, carried out at the regional level since international donors were the main funders of the programme and their offices were established at the regional level.

### Challenges of including PMTCT activities in district health plans

Despite the decentralised management of the PMTCT programme, the informants at the district, regional and health facility levels shared their frustration, as they explained that their PMTCT-related priorities were rarely taken into account in the final plans meant for actual implementation. The main challenge, they pointed out, was the central roles of the donor and of the MoHSW in the prioritisation processes, on the one hand, and the inherent weaknesses in the proceedings of the District Health Team on the other. We shall look at these findings in some detail below, starting with the district informants’ experience, the level located at the heart of Tanzania’s decentralisation policy.

#### Donor influence over the district health plans

The involvement of the region in the review of the prioritised activities in the CCHP was perceived by all the district informants as a serious barrier to the accommodation of their own prioritised PMTCT-related activities. District informants raised concerns that activities that were included in the Council Comprehensive Health Plan would not necessarily guarantee that they would be honoured by the regional secretariat. It was established that this was commonly the case with PMTCT related activities. A district informant explained the reason behind this: *“Our idea was to train more persons from the health facilities providing PMTCT services so that when one staff is not around service provision doesn’t stop. We also planned to provide refresher training in accordance with changes in the guidelines to enable each health facility to provide anti-retroviral combination therapy to the mothers. But the Regional Secretariat cancelled the activities because the PMTCT programme is a priority of the donors (i.e. They were left to them). Last year (2010), we did not have any activity in the Comprehensive Council Health Plan related to PMTCT”* (IDI- district informant).

The challenges that continue being reported in the PMTCT programme from the local levels have prompted district managers to push consistently for the establishment of follow-up mechanisms aimed at improving the quality of the PMTCT services in the many facilities that did offer them. However, the proposed activities were not accommodated in the plan: *“The reported priorities are to improve the quality of the services provided at the health facilities that have already received PMTCT training by making sure that services are provided in accordance with the guidelines. This implies preparing a budget for purchasing HIV diagnostic kits in case facilities run out of stock, and having a budget item to support the transportation of specimen from health facilities to the zonal laboratory and returning the results on time. Now, because the managers at the regional level are the ones making decisions on HIV in general and on PMTCT in particular in collaboration with the donors, you find that the plan is not approved”* (IDI-district informant). The informants explained that the donors’ primary aim was to increase coverage in terms of the number of health facilities providing PMTCT services and trained health care providers to expand their care and treatment services: “*What donors focus on is the coverage of the PMTCT service in terms of number of facilities providing services and trained staff. When we were invited to the region to defend our plan (CCHP) we were told that health care providers at 32 of the 37 health facilities in Mbarali district have already been trained. The remaining five facilities are now included in the donor budget. Consequently our prioritised activities were cancelled”* (IDI-district informant)*.* The district informants also complained that whatever activity they proposed with a view to enhance the PMTCT programme would be cancelled by the regional secretariat as the aims and the priorities had already been made by the donors. The district informants thus reported that they experienced loss of influence over the planning process that they were otherwise supposed to own.

#### Ministry of Health’s influence over the district health plans

District informants expressed a lack of autonomy when reviewing the PMTCT priorities in the district health plans. This experience was not only related to the substantial influence of the donors, but also to the directives coming from the national level. The district informants explained that they received guidelines from the MoHSW relating to the inclusion of nationally-prioritised health interventions. In particular they said that the need to adhere to the demands laid out in the Essential Health Package while attempting to set local priorities proved tricky: “*The challenge in the planning for PMTCT activities is that the district planning team is always faced with a challenge on how to address the requirements of diverse guidelines while simultaneously adhering to the Essential Health Package. There are several groups to consider in each priority area. Taking into consideration our capacity in terms of funds and staff, they found it increasingly difficult to prioritise (some of the locally-relevant issues)”* (IDI-district informant).

In fact, the Regional Secretariat was often in an ad hoc manner, instructed by the MoHSW to include activities in the CCHP that were neither prioritised nor planned for locally. An informant at regional level had this to say: *“In the 2010 financial year we were instructed by the Ministry of Health to include ’Kangaroo Mother Care’ activities’ (i.e. procedures for saving the lives of neonates) during the review of the Comprehensive Council Health Plans. This necessitated the cancellation of other prioritised activities at the district level because the budget ceiling had already been exhausted”* Additionally, informants at the regional level found the restrictions imposed upon them by both the donors and the national authorities as limiting their abilities to manoeuvre independently. In a similar vein, informants at the district level reported that restrictions placed upon them by the regional and donor levels as well as by the nationally established priority areas (including the budget ceilings) limited their potential for independent priority setting and consequent allocation of funds. In practice, such imposition blocked all PMTCT activities prioritised by the district, they explained.

### Challenges pertaining to the inclusion of local PMTCT priorities in the regional plans

Regional informants emphasised that PMTCT activities are usually planned at the programme level which is located at the regional level. This was linked to the fact that international donors had moved their project administration from the national to regional levels in an effort to ‘decentralise’ their activities and to work more closely with the regional teams. Although the donors had been instructed by the MoHSW to respond to the regional and district plans and priorities, the informants lamented that the donors did not follow these instructions, and the regional administration was not in a position to halt the donors’ continued follow up of their own globally defined priority areas. Despite being aware of their obligation to ensure the development of the regional PMTCT plans, the regional team found it increasingly difficult to intervene as they remained mindful to reflect the donors’ priorities: *“Donors have their own priority areas and they do stick to them. For example, Walter Reed is concentrating on care and treatment alone. It is difficult to include our local priorities,”* (IDI- regional informant). Regional managers were moreover given strict budget ceilings by the donors, ceilings that hindered them from expanding or modifying the scope of the activities during the planning phase. The regional managers for example expressed their dissatisfaction with the lack of preventive effort in the plans executed. One regional informant said that they were so alarmed by this anomaly that they asked donors directly: “*If you are focusing on care and treatment alone while individuals are continuing to get infected with HIV, we are doing nothing’” (*IDI-regional informant)*.*

In a few cases, the informants explained that the donors would allow regional managers to include their own priorities in the plan after they had received substantial complaints: “*This financial year (2010–2011) donors brought a ceiling of 10 million Tanzania shillings and they wanted us to train health workers on male involvement in PMTCT. We said “no”- we have no problem with the health workers as they have already been trained on PMTCT. We don’t need to invest again in the health workers at this point. Let’s invest in community sensitisation so that we can talk to community leaders about the importance of the PMTCT programme; how to prevent HIV from infecting the children, etc”* (IDI-regional informant). The donors were said to have eventually warmed to this suggestion, paving way for planning of several community meetings with the same 10 million Tanzania shillings budget ceiling. The regional managers clarified that since the PMTCT programme is managed at the regional level, the districts do not receive funds to carry out these activities. Instead, it is the regions that handle funds for implementing PMTCT-related activities: “*If it is training, the district is told to bring the participants (to the regional level); if it is supplies we at the regional level purchase everything and send the same to them* (IDI-regional informant)*.* Regional informants expressed that when district priorities are not met they do encourage the districts to use the money collected from ‘cost- sharing’ (collected from user-fees and Community Health Funds) to implement the priority areas presented by the health facilities.

#### Administrative confusion

Confusion emerged among district informants regarding who was actually involved in the processes of setting priorities related to PMTCT. Although the regional managers claimed that they had sought inputs from the district in the PMTCT planning processes, this was strongly refuted by the district informants. Key individuals managing the PMTCT programme at the district level explained that they had never been asked for their opinions or priorities when the regional plan was being developed, nor did they receive a copy of the approved plan. In practice they explained that they were merely ‘receiving instructions’ , primarily in terms of PMTCT coverage: *“The regional staff may visit the district and ask ‘how many health facilities are not providing PMTCT services’. You respond – ‘five facilities’. You are then asked, ‘Give us their names’. Once you provide the names that becomes the end of the business” (*IDI-district informant). The district perceived the regional level to act according to the donors’ wishes and demands without involving relevant stakeholders at the lower levels, hence overlooking the overriding interests at the grassroots level.

### Challenges of the district health plan team

#### Experiences from the health facility informants over the planning processes

The findings from the interviews indicated moreover that there were challenges beyond the donor/regional/national vs. district dynamics that had implications for local priority setting processes in relation to the PMTCT programme. Key stakeholders at the health facility level found that, although they were the ones with hands-on experience with the programme, their experiences and views were not taken into account by the district planning team, and they rarely surfaced in local discussions, plans or in budgets relating to the PMTCT programme. Health facility staff participating in the FGDs revealed substantial frustrations about this situation. One participant recounted how the PMTCT focal person at the hospital level had prepared a detailed list of prioritised PMTCT activities after being asked to do so. The list was then submitted to the district HIV/AIDS coordinator, but she said: *“I have noticed that during the planning sessions we are always asked to bring in our priorities. We as hospital staff therefore said, ‘Let’s prioritise training so that each staff who works under the Reproductive and Child Health Department becomes aware of PMTCT and how to take care of an infected mother”* (FGD-participant, health facility). As was often the norm, their main priority area, in this case, the training of staff was not accommodated in the eventual plan.

Other informants from health facilities similarly emphasized the lack of inclusion of their priorities and explained the feelings of frustrations among staff due to that development:*“*W*hen someone from a PMTCT programme requests the inclusion of a certain activity in the plan, it is because s/he [‘ana’ in Swahili is a gender neutral prefix] knows its importance. The problem with the planning team is that some members don’t know the importance of the activities, so they don’t prioritise them. For instance, in the current plan, when the district planning team came back from the planning meeting, the facility staff asked-‘If we had identified our priorities and sent them to the planning team, and later we saw that none of our priorities was taken on board… Would it then make sense to involve us?’ If managers ignore our activities, then what is our role in the planning?” (*IDI-health facility informant)*.* Health facility informants also questioned the rationale of involving normal staff in identifying their priorities and submitting them to the higher level when at the end these suggestions are never considered in the plans. They insisted that the planning team included only activities that they themselves believed were important without even providing proper justifications.

#### Seeking inputs from facility staff during planning processes at the district level

None of the PMTCT in-charges, except at the hospital level, had been asked to bring in their priorities related to PMTCT. The health personnel did not even know the source of funds that were used to implement the PMTCT activities and found themselves far removed from the priority setting and decision-making bodies. The uncertainty about the funding and priority setting situation strongly emerged in the FGDs, particularly at the Faith-based hospital. The group participants expressed that they were not aware of what was going on regarding general priority setting even though they were aware that, as a faith-based facility they should get a certain allocation from the district budget: “*May be we are not asked to bring our priorities because we are located at a mission hospital so we are just waiting to be informed by the district on what to do”* (FGD-health facility). The FGD participants at the district hospital on the other hand, were somewhat more aware of the priority setting processes. Nevertheless, none of the staff who participated in the FGDs or IDIs was aware of the regional health plan which specifically targets the PMTCT programme. A majority of the PMTCT facility in-charges would readily present their own thoughts about the challenges and their priorities related to PMTCT, but they insisted that they were not asked by the district to present them. Among the many critical areas brought up during our discussions included the need for funds for: refresher training on how to fill in the PMTCT forms and on how to carry out basic PMTCT- related tasks; follow-ups of mothers who drop out of the PMTCT programme, the purchase of waste bins for keeping of highly infectious materials; the purchase of sufficient HIV test kits; renovation of the counselling room to enhance privacy; continuous community education; and strengthening of male involvement.

Health facility informants explained that the priorities especially at the maternity section where safe delivery is encouraged and expected as per PMTCT guidelines, were rarely considered. When a few PMTCT-related items were included, they were regularly purchased in an insufficient amounts. As a result several health facility workers reported about the use of personal funds to purchase important items such as dishes and waste bins. Generally, as the participant explained, there was a discrepancy between what they wanted and what was actually implemented.

#### Knowledge and communication gaps

A planning session is held annually at district level in Tanzania. At this meeting, prioritisations and allocation of resources take place on health-related activities, leading to the production of the CCHP. Despite the fact that the district members attending this planning session were educated at the diploma level and above, substantial weaknesses were revealed in terms of a lack of knowledge on how to properly plan employing the allocated ceilings. Challenges of communication between the diverse sections within the district and between diverse administrative levels also surfaced. The communication gap that was found between the people in charge of the PMTCT activities and the representatives of the relevant department during the planning sessions surfaced in many of the interviews. A regional level informant explained: *“In the district health plan, it becomes difficult to accommodate activities related to PMTCT because the PMTCT coordinator does not participate in the planning; rather he/she is represented. There may be a communication gap between them. Since the district health plan accommodates a lot of activities you find that each member is struggling for the inclusion of his/her activities”* (IDI-regional informant)*.* The implication of this statement was again that very few PMTCT activities were taken on board due to budgetary constraints*.* The challenging organisational structure of the PMTCT programme further complicated the process of including priorities found meaningful at the local level: *“The programme coordinator, for example, does not include PMTCT activities such as budgeting for diagnostic kits, medicines, delivery kits and delivery bed in the plan. If you ask her, she would tell you ‘Wait for the pharmacy budgeting’ , and she will only budget for training. As a result, when it reaches the time for pharmacy budgeting I budget for general supplies and drugs”* (IDI- district informant). Generally, what emerged was a confusing structural landscape, which compounded the already existing challenges of incorporating local priorities.

## Discussion

We started out this paper by presenting Tanzania’s commitment to promote decentralised health care, a system in which decision-making and priority setting processes have been devolved to the district level to ensure local level prioritisation, control and accountability [9]. Whereas the decentralisation policy advocates for local planning processes that start at the grass-roots level and move up through the system, this ideal was hardly reflected in the findings of our study related to the PMTCT programme. Neither the district nor regional health plans were seen to incorporate the views and priorities of the categories of people most closely and strategically placed within the PMTCT programme. In spite of the existing structures and the clearly spelled out ideals of decentralisation, we found that in the case of the PMTCT programme the system remained heavily reliant on external funders who tend to guard their own globally generated priority agendas in a manner that disregards local experiences and priorities. Also the district’s dependency on basket funding with minimal internal revenues accruing from within the district has amplified the burden of non-inclusion of local priorities in the annual health plans.

In response to the changes, the donors who support the PMTCT interventions have made attempts at moving closer to the people by relocating their operational base from the national to the regional level. In other words they have shifted their arenas from the international, to the national and more recently to the regional levels. Although presently they work with the regional managers of the programme, the regional administrative level in Tanzania is still far removed from local communities and health facilities. According to the governmental structure the role of the region is to translate policy guidelines from the national level, advise and supervise districts, and review the district health plans to ensure conformity with the national guidelines before they are approved by the District Full Council [8]. In this regard, the donor devolvement to the ‘local’ level appears to have failed to ease and facilitate the inclusion of the priorities identified by the districts in the PMTCT-related activities, when this level is at the heart of the grassroots-based priority setting and decision-making in the Tanzania’s decentralised system. The donors operating in the PMTCT field, according to this study seem to be fairly rigidly sticking to their own globally-defined set of priority areas with little or no willingness to include the district’s experienced demands. The dynamics at work related to priority setting in PMTCT thus seem to operate in a fashion that remain far-removed from the staff at the health facilities, the district administrators and, apparently, even from the programme managers at the regional level.

The PMTCT services in Tanzania have recently been fully integrated into routine RCH services [23], according to the 2011 PMTCT guidelines. It does however seem to remain extremely difficult to locate and coordinate where, when and in what ways the PMTCT activities should be integrated in the planning process within an extremely complex reproductive health package. The findings moreover add substance to the experience that the integration of the PMTCT services into the RCH services has revealed little success in terms of integrated planning, since health planners operate with the (very real) perception that the PMTCT programme is donor-driven and donor-funded. The implication is that the programme is left to others and consequently receives little attention within the complex priority setting processes that surrounds the production of the annual CCHP. An important assessment study of the performance of the health sector reforms and of decentralisation revealed a similar scenario: programmes dealing with diseases that were perceived to be located under ‘vertical programmes’ and thus under donor funding received far less attention during processes of prioritisation as they were perceived to be already catered for, the implications being that local priorities were often not incorporated [15]. In the present study, little knowledge on how to plan properly using the ceiling allocated in a diverse set of priorities, communication gaps between representatives attending the planning sessions and PMTCT in-charges, and lack of interdepartmental collaboration have added to this difficulty and has made it challenging to carry out a meaningful prioritisation process in the PMTCT programme. It has previously been documented that challenges of conflicting personal interests coupled with poor interpretation and implementation of the guidelines are other aspects that may undermine the priority setting processes and overall performances of the district health planning teams [15].

Although the districts have been given the autonomy to prepare and implement their health plans, the Ministry of Health retains a central role in developing policies to be implemented at the local levels in the decentralised health care system [5]. In practice, however, the MoHSW in this particular case seems to continue producing policy guidelines that are perceived as ‘must’ or ‘orders’ by the lower levels of administration. Although the basket funds are remitted to the districts, the planning teams are also in this case given guidelines to follow in their allocations implying: 5-10% (community initiatives), 15-20% (health centre), 10-15% (voluntary agency hospital), 25-35% (council hospital), and 15-20% (office of the DMO) [8]. Thus the guidelines that may be helpful in guiding the process end up limiting the planning team’s capacity to plan as they would wish since the above distribution has to take precedence over all other additional items. Sometimes the ministry officials would ask for the inclusion of activities in the district health plans that are not of local priority. Members of the secretariat at the regional level, who review the CCHP for conformity with the national guidelines, would thus in this case e.g. cancel PMTCT-related activities that were initially planned for in order to accommodate national demands. These national demands are again often generated by global policy and funding bodies such as the UN system and become demands that nations find hard to refrain from. Daniels, the scholar behind the Accountability for Reasonableness Framework for priority setting, has argued that to improve fairness, the planning teams need to work like ‘a football team’ where all players work together for the common goal [30]. Under this notion, activities perceived to be of important will receive attention in the prioritisation process regardless of whether they are from the vertical programme or not, or from lower level staff or not. In fact improved priority setting decisions improve the quality of service provision; they improve stakeholders’ satisfaction, and reduce complaints, thus enhancing trust and proper allocation of resources. The REACT project from which this sub-project emerged has reported positive results pertaining to stakeholder involvement in processes of priority setting, as documented by Maluka for example [31]. However, more studies are deemed necessary to assess reasonably the status of priority setting processes within diverse health related programmes in this and other districts in order to draw broader conclusions.

The scenario that has emerged in the present study is challenging as it seems to question the legitimacy of the priority setting processes pertaining to the PMTCT programme as exercised in the country. Moreover, it asks questions on the manner in which the ideals behind decentralisation are fulfilled. The findings of the present study add to existing evidence of a continuation of top-down and external influence, whether donor or governmental documented in other studies [16,32]. Johansson [32] has, for example, in his study on Tanzania revealed how priorities are set by international and national managers pertaining to the project related to the eligibility criteria for receiving antiretroviral drugs, a scenario which implies that lower level actors from where implementation takes place had no room to contribute. Other studies have focused on the challenges in priority setting processes at the district level [11,16,27]; indicating that health facility and community views are rarely taken into consideration, that no clear procedures are followed and that there are no clearly spelled out roles for the different committees at the district and health facility levels, rendering the health committees redundant and inactive. Weak health information systems, moreover, hinder the availability of credible and reliable evidence required by the District Health Plan team at the time of setting priorities [10,33], a situation which in turn makes it difficult to make meaningful prioritisation. The involvement of lower levels in the planning process should be aimed at ensuring that resources are targeted to those in need. On this point, the Council on Health Research for Development (COHRED) asserts, ‘The ones who own the problem are the ones who can provide the solutions’ , and they need to actively participate in setting priorities [34].

In principle, one must be also careful when indicating that the study findings are relevant beyond the field we have focused on. However, we do wish to suggest that the findings of the present study together with similar findings from other studies [11,16] indicate that the decentralisation policy seem to work to reinforce the established power structures rather than integrating priorities of lower levels stakeholders which was aimed at with the reform. It is also important to keep in mind that colonial legacies and customary power structures lead lower level staff to fear open disagreement, making it difficult for them to meaningfully execute the authority granted to them. This tendency creates a new form of dependency on the donors/ foreign experts and other higher ranking individuals, creating scenarios where local officers remain locked in a system over which they have little control.

In this particular case, the continued donor-dependence has contributed to the continuity of a top-down approach where PMTCT managers feel that they have little option but to adhere to the donor’s priorities, hence they experience a loss of autonomy. In the past few years the PMTCT related priority areas of the donors have been to improve access in terms of coverage of services [35]. This has been a worthy contribution with tangible results in a vast numbers of African countries. The PMTCT services are today available in most health facilities with RCH services in Tanzania [21], and at least a single member of staff has been trained in each facility providing the services. These measures however, do not ensure the quality of the programme which continues to experience severe challenges [26,36]. Moreover, the health systems challenges continue to hamper its successes and raise concerns over the quality of the services on offer. Similar findings were reported by Johansson from another rural district in Tanzania [32].

On the whole, effective implementation of sector-wide approaches where donors support the budget of the health sector through basket funding emerges as a useful way to enable districts to identify their own priorities [37]. These approaches, when effectively implemented, can facilitate the smooth implementation of the decentralisation policy as it can allow for a shift from vertically-focused health programmes and centrally-controlled budgets to more comprehensive health planning and locally-controlled health budget structures at the district level. Such approaches, moreover, are aimed at reducing external power influences [38]. In this study these aims do not seem to be fulfilled in the case of the PMTCT programme. The claim that funds were inadequate could result from a lack of readiness to allocate the budget items to the programmes perceived to be donor-driven or could stem from a lack of experience and capacity to prepare or implement the plan. A study by Semali [12] revealed that there might be opposition to the transfer of authority by district stakeholders by giving less priority to vertical programmes activities even when they are integrated into the horizontal services. Despite these challenges, it is important to note that improving the implementation of the decentralisation policy entails eliciting values and criteria for priority setting from lower level stakeholders in the health care system. In this effort, special attention should be paid to the methods to be used in bringing up local priorities. Only a continuous concern with the dynamics at work in the health systems will allow for continued pressures to be kept on local authorities in a manner likely to facilitate increasingly fair and inclusive priority setting processes whether in PMTCT or other health programmes. When priority setting processes are grounded in the local context, it is more likely that the decisions reached will be perceived as relevant by the stakeholders, and that decisions will ultimately be experienced as improving the quality of health services on offer, which should be the main outcome of a fair priority setting process in a decentralised health care system.

### Study strengths and limitations

This study is based on a single rural district. As such it is hardly possible to generalise its findings to the rest of the Tanzanian districts. Nevertheless, the emphasis placed on donor funding of the PMTCT programmes and the integration of the activities in the CCHP is a national policy. Thus, it is likely that dilemmas of priority setting related to the continued central role of the donors and of MoHSW, and the challenges linked to the weaknesses inherent in the district planning team that were found in this study as hampering the effective implementation of the decentralisation policy can be of some relevance also in other districts in the country.

## Conclusion

The priority setting processes related to the prevention of the mother to child transmission of HIV programme demonstrate substantial weaknesses, weaknesses that challenge the principles of Tanzania’s decentralisation policy where principles of bringing local priorities up-front are located at the core. The findings of this study indicate that the strong donor influence coupled with the MoHSW’s high profile role in the country’s priority setting facilitate the continuity of the top-down approaches that were supposed to have been eliminated through the promotion of the participatory, grassroots-based bottom-up approaches. There is an urgent need to streamline the present strategies and aims of the decentralisation policy at all levels of the health care system to ensure that they are responsive to actual grassroots needs. Particular focus should be placed on the problematic continuation of undue influence and perpetuation of the top down approaches by donors and the Ministry of Health to ensure that the planning and prioritisation processes follow laid-down procedures and guidelines with the overall aim of improving health service delivery in a manner that is perceived as relevant and fair to the populace, particularly at the grassroots level.

## Abbreviations

CCHP: Comprehensive Council Health Plan; CHMT: Council Health Management Team; DHBs: District Health Boards; FGDs: Focus Group Discussions; IDIs: In-depth interviews; MoHSW: Ministry of Health and Social Welfare; PMTCT: Prevention of Mother to Child Transmission of HIV; RCH: Reproductive and Child Health.

## Competing interest

The authors declare that they have no competing interests.

## Authors’ contributions

EHS conceived the idea of the project in collaboration with AB, collected the data, analysed and drafted the manuscript. AB took part in the analysis of the data. AB and LEGM reviewed and provided substantial inputs to several drafts of the article. All the authors have read and approved the final manuscript.

## Pre-publication history

The pre-publication history for this paper can be accessed here:

http://www.biomedcentral.com/1472-6963/13/273/prepub
